# Highly Stable Cycling of Silicon-Nanographite Aerogel-Based Anode for Lithium-Ion Batteries

**DOI:** 10.1021/acsomega.0c05214

**Published:** 2021-03-01

**Authors:** Rohan Patil, Manisha Phadatare, Nicklas Blomquist, Jonas Örtegren, Magnus Hummelgård, Jagruti Meshram, Deepak Dubal, Håkan Olin

**Affiliations:** † Department of Natural Sciences, Mid Sweden University, Sundsvall 852 30, Sweden; ‡ Centre for Interdisciplinary Research, D.Y. Patil Education Society (Deemed University), Kolhapur, Maharashtra 416006, India; § Centre for Materials Science, Queensland University of Technology (QUT), 2 George Street, Brisbane 4000, Australia; ∥ School of Chemistry and Physics, Science and Engineering Faculty, Queensland University of Technology (QUT), Brisbane 4000, Australia

## Abstract

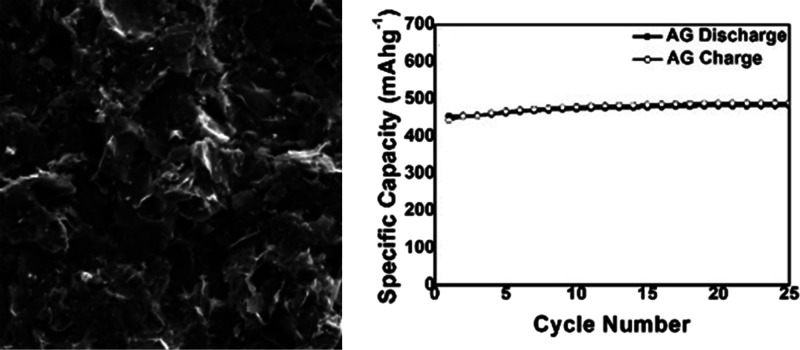

Silicon anodes are considered as promising electrode materials for next-generation high capacity lithium-ion batteries (LIBs). However, the capacity fading due to the large volume changes (∼300%) of silicon particles during the charge–discharge cycles is still a bottleneck. The volume changes of silicon lead to a fracture of the silicon particles, resulting in recurrent formation of a solid electrolyte interface (SEI) layer, leading to poor capacity retention and short cycle life. Nanometer-scaled silicon particles are the favorable anode material to reduce some of the problems related to the volume changes, but problems related to SEI layer formation still need to be addressed. Herein, we address these issues by developing a composite anode material comprising silicon nanoparticles and nanographite. The method developed is simple, cost-efficient, and based on an aerogel process. The electrodes produced by this aerogel fabrication route formed a stable SEI layer and showed high specific capacity and improved cyclability even at high current rates. The capacity retentions were 92 and 72% of the initial specific capacity at the 171st and the 500th cycle, respectively.

## 1 Introduction

Lithium-ion batteries (LIBs) are gaining much research interest in portables devices and electric vehicles because of their high energy density and long cycle life. Most of the commercially available LIBs use graphite as an anode material. However, these graphite anodes cannot meet the ever-increasing demand of high energy density due to their limited theoretical specific capacity of 372 mAh g^–1^.^1−3^ Among various materials, silicon is an attractive anode material for LIBs due to its high specific capacity of 4200 mAh g^–1^, which is more than 10 times higher than that of graphite.

Although silicon anodes deliver high capacity for LIBs, they normally suffer from poor cycling stability due to the large volume changes during the charge–discharge cycles.^4,5^ Repeated volume fluctuations cause fracture and pulverization of the silicon particles, leading to repeated formation of a solid electrolyte interface (SEI) layer on the surface. The formation of the SEI layer causes successive accumulation of the electrolyte that leads to loss of conductivity and fade in capacity with cycling. Various strategies have been applied to overcome this problem, including formation of silicon nanoparticles, silicon nanotubes, porous structures, etc.^6−9^ Nanometer-scaled silicon particles are preferred over micron-sized Si particles since nanoparticles significantly improve the cycling performance of anodes.^7,10,11^ Nanostructures are expected to enhance the electrochemical performance of Si anodes as the reduced size of Si particles allows greater alloying/dealloying rates. In addition, the volume fluctuations can also be shielded after reducing the Si particles to nanosize. The cracking of silicon particles is largely reduced by using nanostructured silicon, thus achieving good material stability.^5^ However, nanosized particles have numerous challenges, for instance, high surface area, high production cost, and handling difficulties.^12−14^ The high surface area linked with the nanoparticle size may increase the unwanted electrolyte reactions leading to the SEI layer formation on the surface. The continual growth of the SEI layer will gradually deplete the available Li^+^ ions and also reduce the amount of the electrolyte. The consequence is the degradation of overall performance, which leads to poor cycling life, self-discharge, and highly irreversible capacity. It further leads to high resistance between the particles. Although various strategies were used for synthesizing silicon nanoparticles, nanowires, and nanospheres, the degradation of specific capacity in the initial cycles and the scalability of the material synthesis are the major issues with these strategies.^9,10,15−19^ Hence, to obtain stable SEI, it is necessary to design an electrode material that has the benefits of a nanostructure but avoids the manufacturing complexity associated with it.

Herein, we have developed Si-nanographite aerogel as a potential anode material for next-generation LIBs by the simple and cost-efficient aerogel fabrication method.^4^ In this work, larger silicon particles are replaced with nanosized silicon particles because even though we achieved good specific capacity in our previous study^4^ using larger silicon particles (fraction of these particles are converted to nanosized silicon particles via thermal heating), the rapid degradation of specific capacity at later cycles was still a major concern. The rapid degradation of specific capacity is mainly attributed to the fracture of larger silicon particles. Nanosized silicon powder may give less particle fracture and a more stable performance. Further, polyvinyl alcohol is replaced with natural polymer sodium alginate as compared to our previous study.^4^ Sodium alginate is naturally occurring, less toxic, and a low-cost polymer compared to polyvinyl alcohol. Another motivation behind changing the parameters is to test the feasibility of the aerogel fabrication method for a different scenario. The aerogel-based electrode with changed parameters shows formation of a stable SEI layer that enhanced the electrode stability. The nanocomposite electrode delivered a 453.8 mAh g^–1^ capacity, and the capacity retentions were 92 and 72% of the initial specific capacity at the 171st and the 500th cycle, respectively.

## 2 Experimental Procedure

### 2.1 Materials and Methods

Sodium alginate and Si powder (particle size ∼100 nm) were obtained from Sigma Aldrich. Nanographite (NG) was produced from thermally expanded graphite (EXG 9840, Graphit Kropfmühl, Germany) by the process portrayed before,^20−22^ without any changes. Sodium alginate is a characteristic polysaccharide product, a solvent in both cold and hot water with solid agitation and can consolidate and tie and has the capacity to form a porous structure.^23^ The characteristic structure of alginate can successfully avoid the accumulation of Si NPs during aerogel preparation and improve the SEI stability.^24^

The Si-nanographite aerogel structure was prepared in the same way as described in the previous article except that the 2 wt % polyvinyl alcohol solution was replaced with 1.5 wt % sodium alginate solution, and silicon nanoparticles (particle size ∼100 nm) were used instead of microparticles.^4^ Si (0.125 g) was added in the 100 mL sodium alginate solution with addition of 0.5 g of nanographite.

### 2.2 Material Characterization

Thermogravimetric analysis (TGA) of aerogel (AG), silicon, and nanographite was accomplished using a Mettler Toledo TGA-1. The silicon weight percentage in the AG structure was determined by TGA analysis. Crystallographic information was studied in the 2θ range from 10 to 80° by the X-ray diffraction technique (XRD; Bruker D2 phaser) with Cu-Kα (λ = 1.54184 Å) radiation. The morphologies and structural characterization of the AG structure and electrodes were studied using a field emission scanning electron microscope at 5 kV (MAIA3, TESCAN).

### 2.3 Electrochemical Measurement

The electrodes were prepared by making a slurry of an AG, nanographite, and sodium alginate binder in a weight ratio of 6:3:1 in distilled water. To form the slurry, mixture was strenuously stirred using Ultra-Turrax T25 at 10,000 rpm for 60 min, engaging an S 25 N-10 G shear head. The slurry was then coated on copper foil (1 mg cm^–2^) (label: AG electrode). The half-cells were assembled in a glove box under an argon atmosphere using AG or NG as working electrode and lithium metal foil as a reference electrode in LP40 (1 M LiPF_6_ in a mixture of ethylene carbonate (EC) and diethyl carbonate (DEC) in a 1:1 weight ratio) as an electrolyte with a Celgard 2325 separator. Cyclic voltammetry (CV) was performed on a VersaSTAT 4 in the potential range of 0.01–2.0 V (vs Li/Li^+^) at a scan rate of 0.1 mV s^–1^. The galvanostatic charge–discharge performance was between 0 and 1.5 V (vs Li/Li^+^) using a LabVIEW-based PXI system. Electrochemical impedance spectroscopy (EIS) measurements were performed between 1 MHz and 0.01 Hz with an amplitude of 10 mV.

## 3 Results and Discussion

### 3.1 Materials Analysis

**Figure 1:**
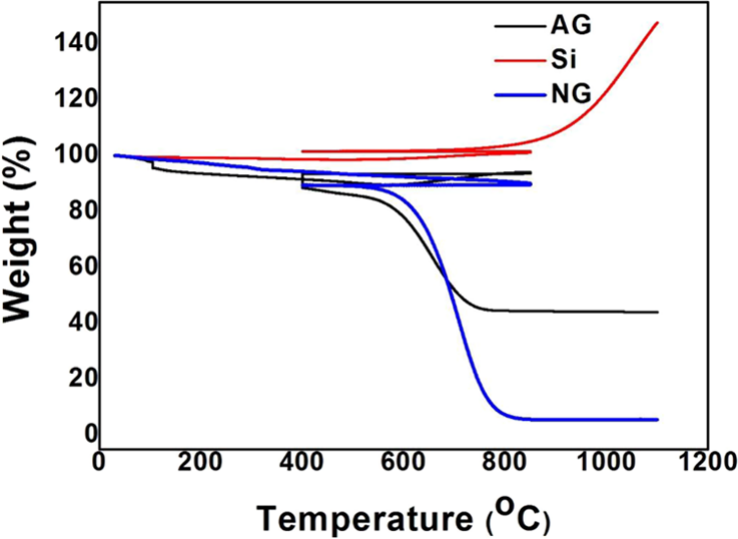
TGA profiles of AG, silicon, and NG.

TGA investigation was carried out for silicon and nanographite to compute the amount of silicon in the AG structure. The analysis was performed for AG, silicon, and nanographite by heating the samples in a nitrogen atmosphere from 30 to 850 °C followed by cooling from 850 to 400 °C at a pace of 20 °C/min with a 10 min isotherm at 400 °C. Thereafter, the atmosphere was switched to an oxygen atmosphere. In the oxygen atmosphere, the material was heated from 400 to 1100 °C at a rate of 20 °C/min TGA curves for silicon, nanographite, and AG, as depicted in Figure 1. It is revealed that the silicon sample gains weight in the oxygen atmosphere starting from about 700 °C, representing the oxidation of silicon. The AG sample in the nitrogen atmosphere demonstrates no significant weight loss, while in the oxygen atmosphere, it loses weight by 55.50% from 400 to 800 °C, which can be assigned to the combustion of graphite. A similar trend is also observed for the NG sample. The weight of sodium in the AG sample was calculated based on the initial weight of sodium alginate. The TGA curve of Si and NG at 1000 °C indicates that Si gains weight by 28.09% due to oxidation, while NG undergoes combustion with a remnant of 5.16%. The combined weight of silicon and nanographite is estimated based on these results while considering sodium as stable. The results are presented in Table 1. The weights of the total AG sample, which are silicon, sodium, and nanographite, were 15.93, 20.34, and 63.73% respectively. The AG, nanographite, and sodium alginate binder were mixed to form the final electrode at a weight ratio of 60:30:10. The final weights of silicon, sodium, and nanographite in the AG electrode were 9.55, 12.19, and 68.23%, respectively.

**Table 1:** Weight Analysis of Silicon, Sodium, and Nanographite in the AG Structure from TGA

	weight from TGA at 1000 °C [%]	estimated weight [%]
silicon	128.09	15.93
sodium	100	20.34
NG	5.15	63.73
AG	44.03	100

**Figure 2:**
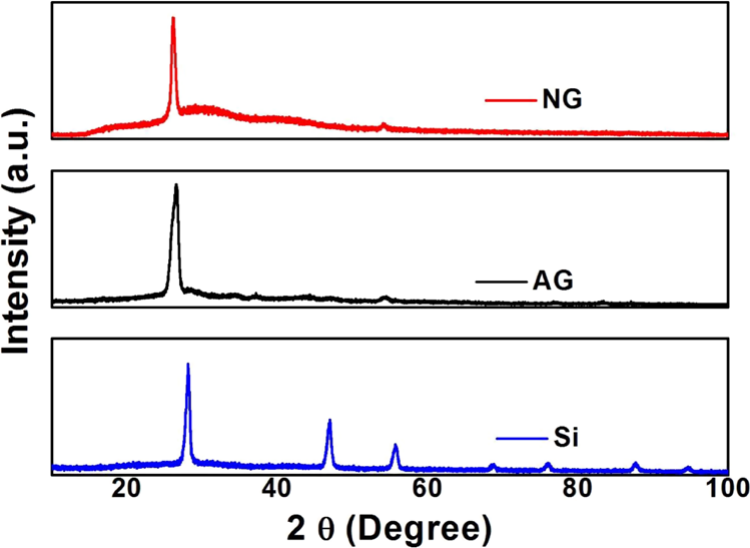
XRD patterns of Si, NG, and AG.

XRD patterns of NG, AG, and Si are shown in Figure 2. Major diffraction peaks are observed at 28.3, 47.0, and 55.8° for Si corresponding to (111), (220), and (311) lattice planes, respectively.^4^ The peak seen at 26.1° of NG is related to the (002) lattice plane of graphite.^25^ In the XRD pattern of AG, a diffraction peak corresponding to the (002) plane of carbon/graphite is observed at 26.1°, which indicates that the Si-nanographite matrix is surrounded by a thick layer of carbon aggregated due to the residue remaining after heat treatment. This layer of carbon suppresses the peaks of silicon resulting in only one diffraction peak corresponding to carbon/graphite.^4^

**Figure 3:**
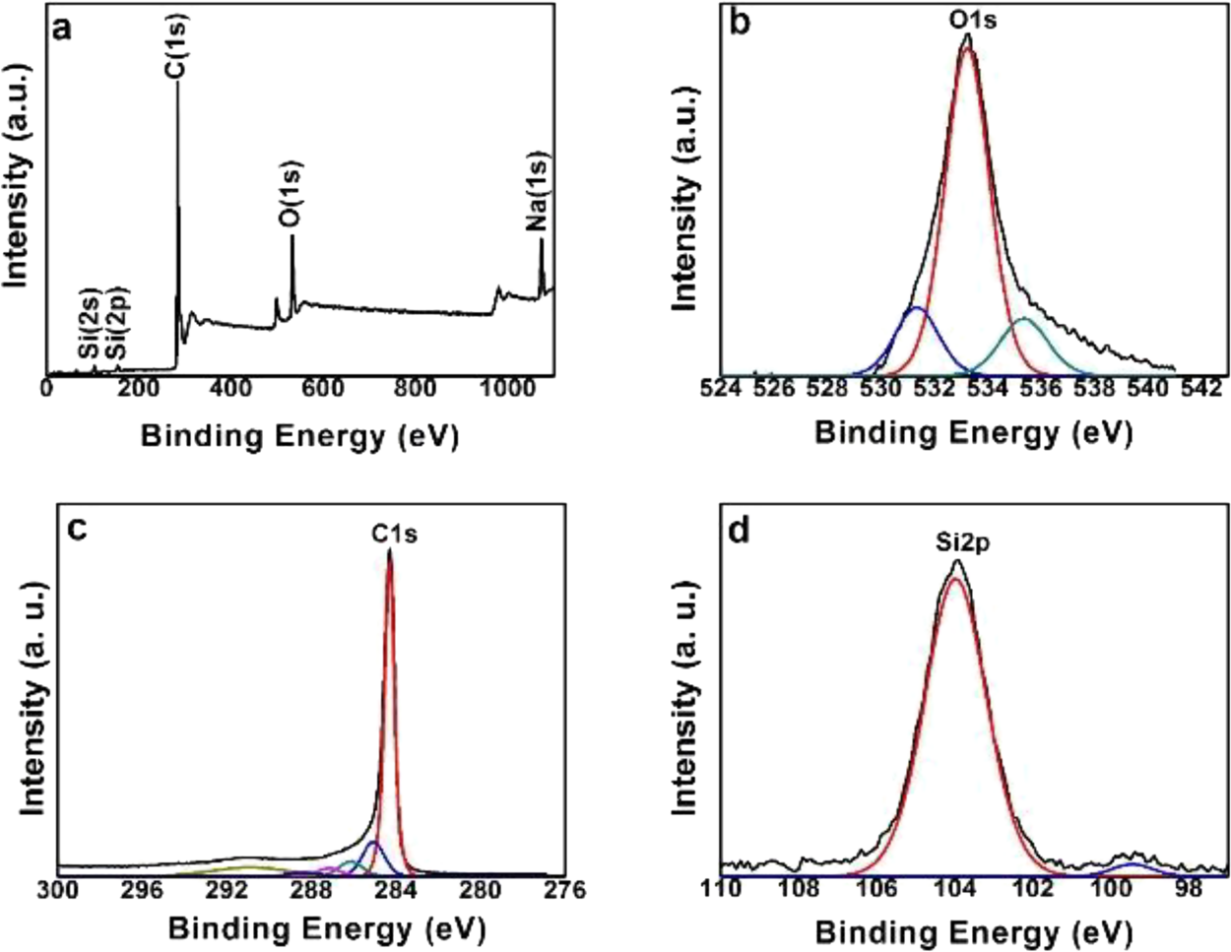
(a) XPS spectrum of AG and (b) O1s, (c) C1s, and (d) Si2p spectra of AG.

The X-ray photoelectron spectroscopy (XPS) spectrum of AG is shown in Figure 3a, while the O1s, C1s, and Si2p spectra of AG are shown in Figure 3b–d, respectively. The O1s peak is observed around 533 eV because elements like Si, Na, and C undergo surface oxidation. This core scan of O1s shown in Figure 3b can be fitted by three excitation peaks at 531.3, 533.2, and 535.4 eV related to Na–O, C–OH/Si=O, and π–π* excitation, respectively. A strong peak at 284.3 eV of C1s (Figure 3c) indicates C–C sp^2^.^8^ The XPS spectrum of AG shows the peaks at 104 and 163.5, which might correspond to Si2p and Si2s, respectively. Two peaks can be seen from the core spectrum of Si2p. The peak at 99.4 eV occurs due to elemental silicon, and the one at 104 eV comes from Si^4+^.^26^ The peak around 1073 eV is related to Na1s. A peak of Si–C is absent in the spectrum, indicating that there is no formation of SiC during the formation of aerogel.

### 3.2 Electrochemical Performance Evaluation

**Figure 4:**
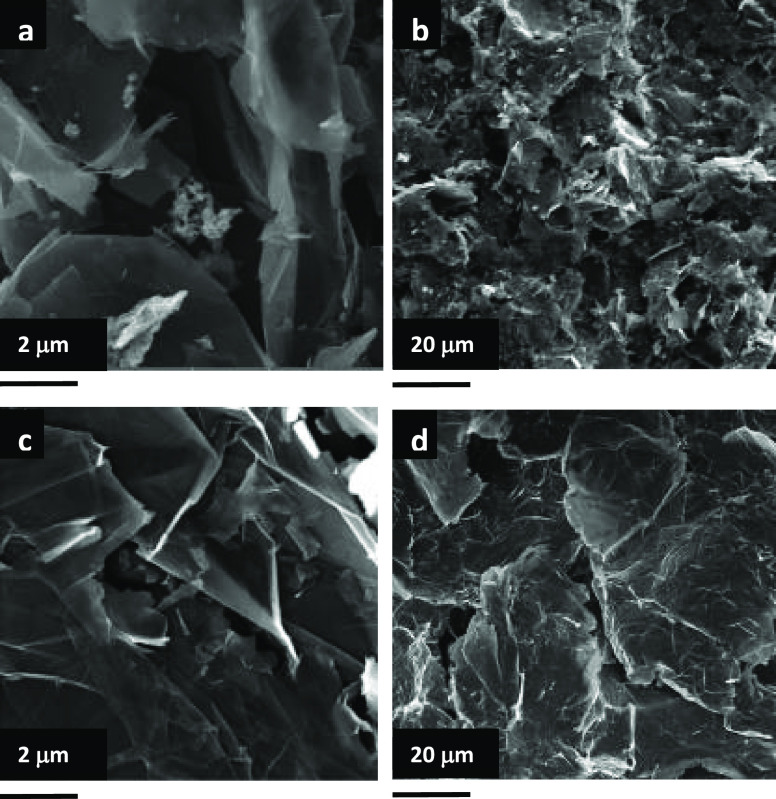
SEM images of the AG (a, b) and NG electrodes (c, d).

Morphologies of the AG and NG electrodes are studied with scanning electron microscopy (SEM), and the corresponding micrographs are shown in Figure 4a,b and Figure 4c,d, respectively. Large amounts of pores of various sizes and relatively small nanographite flakes are observed in the AG electrode, as compared to the NG electrode. In the NG electrode, nanographite flakes of various sizes with relatively few pores are observed. The occurrence of excess pores in the AG electrode can be credited to the porous structure of Si-nanographite aerogels that were formed due to the emission of gases and water vapor during the heating process. The surface of the electrode looks like a microstructure with silicon nanoparticles. Such a microstructure is beneficial in minimizing the interaction of the electrolyte with the electrode and forming the stable SEI layer, leading to the stable performance in terms of capacity.

**Figure 5:**
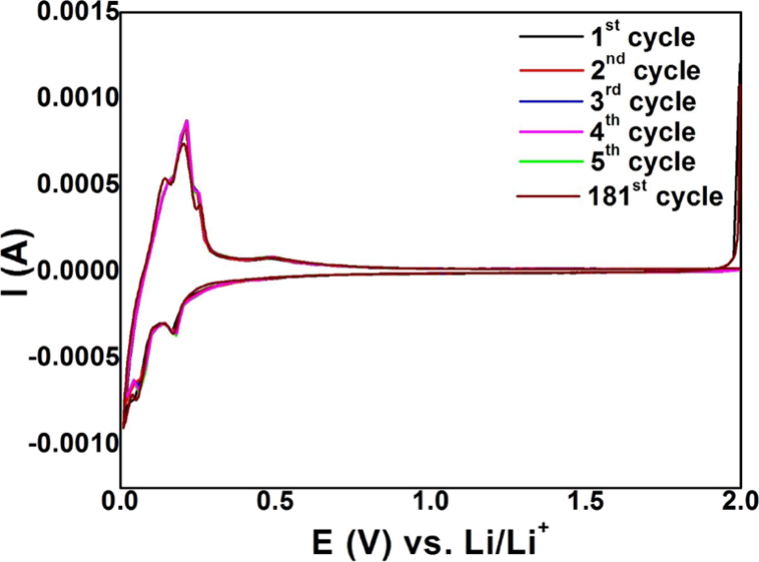
Cyclic voltammograms of the AG electrode at a scan rate of 0.1 mV s^–1^ for the 1st cycle, 2nd cycle, 3rd cycle, 4th cycle, 5th cycle, and 181st cycle.

The redox reactions were studied by CV measurements, as shown in Figure 5. Usually, a peak related to the formation of the SEI layer appears between 0.8 and 0.4 V during the first discharge,^8^ but as seen from Figure 5, no significant peak is observed. The absence of the peak implies the formation of a stable but thin SEI layer on the surfaces of silicon and carbon layers. Two cathodic peaks are observed at 0.16 and 0.01 V for all the curves, while the peak at 0.01 V splits into two distinct peaks in the subsequent cycles. The cathodic peak around 0 V is the characteristic peak for both amorphous and crystalline silicon, while the peak at 0.16 V is related to the conversion of crystalline silicon to an amorphous structure.^8,27^ Two anodic peaks appeared at around 0.21 and 0.49 V for all the cycles, corresponding to the dealloying process of Li–Si alloys.^8^ It is inferred that the formation of an extremely conductive phase and the stable structure of Si enable lithium extraction starting at a relatively low potential, improving the reaction kinetics. It is noticeable that the lithiation and delithiation peaks showed almost the same intensity from the first cycle to the fifth cycle and hence the same area between the curves for increasing and decreasing potential, which can be attributed mainly to the stable silicon structure. The CV curve for the 181st cycle showed peaks at the same positions with similar intensity as that of the first five cycles, signifying the stability of the AG structure. The similarity in the CV curves for different cycles can be explained by the formation of a stable SEI layer. The formation of a stable SEI layer can further be confirmed by impedance spectroscopy results.^27^

**Figure 6:**
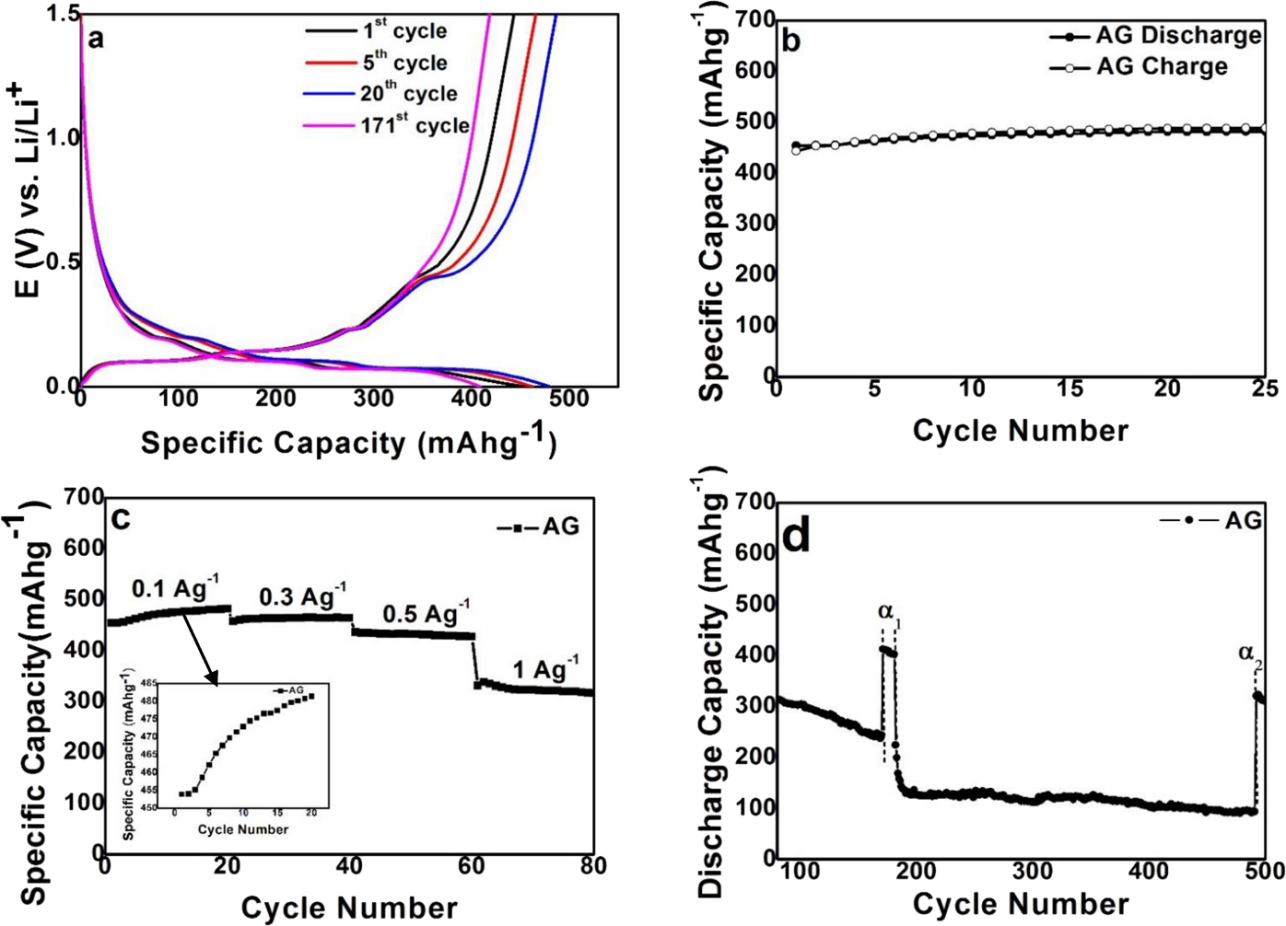
(a) Typical charge–discharge profiles of the AG electrode at the 1st, 5th, 20th, and 171st cycles at a current rate of 0.1 A g^–1^. (b) Specific capacity of the AG electrode at a current rate of 0.1 A g^–1^. (c) Cycling performance of the AG electrode at different current densities and (d) cycling performance of the AG electrode at current rates of 0.1 and 1 A g^–1^ from 80 to 500 cycles.

The charge–discharge profiles of the AG electrode at different cycles at a rate of 0.1 A g^–1^ are shown in Figure 6a. The first discharge cycles showed a slope between 0.16 and 0.01 V, which can be associated with the peaks occurring at a similar voltage during the first lithiation cycle in the CV curve. In addition, it is observed that the plateaus are present in the lithiation region from 0.16 to 0.01 V even up to the 171st cycle, signifying that lithiation reaction occurs in AG without the pulverization of the Si particles and the breakdown of the electrode.^8^ The plateaus witnessed in the regions of 0–0.3 and 0.43–0.6 V in the charge curve relate to the delithiation of the Si–Li alloy to Si. This can be associated with the anodic peaks observed at a similar position in the charging of the first cycle.^8^

Figure 6b shows the cycling performance of the AG electrode at a current rate of 0.1 A g^–1^. The first cycle discharge capacity of the AG electrode is 453 mAh g^–1^. The discharge capacity of the AG electrode has improved from 453.8 to 481.6 mAh g^–1^ from the 1st to the 25th cycle with a 99% Coulombic efficiency. This trend of stable increase in the capacity is unique for the AG electrode. The slight increase in specific capacity can be credited primarily to the improved extraction kinetics in the initial 25 cycles.

The rate performance of the AG electrode at different current densities is shown in Figure 6c. It is observed that the capacity fade for the electrode is minimal at different current densities and shows stable capacity during each discharge. Another fascinating result is that, after the initial fall in the specific capacity, the AG electrode showed a stable increase in specific capacity for subsequent cycles. The rate performance of the AG electrode from the 80th to 500th cycle is shown in Figure 6d. As the current rate was reduced to 0.1 from 1 A g^–1^ (171–181 cycles), 92% of the specific capacity was recovered. A similar experiment was carried out for subsequent cycles (492–500) in which 72% of the specific capacity was recovered, which further confirms the stability of the AG electrode. Even at the higher current densities, a relatively stable capacity was obtained. From the previous study,^4^ it is believed that a fraction of silicon microparticles are condensed and grow as nanoparticles on the nanographite flakes during the aerogel process, thereby contributing to the better performance of the electrode. In this study, where silicon nanoparticles are used instead of microparticles along with sodium alginate instead of polyvinyl alcohol, no such conversion of silicon nanoparticles to a smaller size was observed in the SEM images. However, the microstructured aerogel helps us to reduce the unnecessary/side reactions of the electrolyte and helps us to overcome problems related to the high surface area of nanoparticles. This results in formation of the stable SEI layer that gives the extremely steady performance of the electrode.

**Figure 7:**
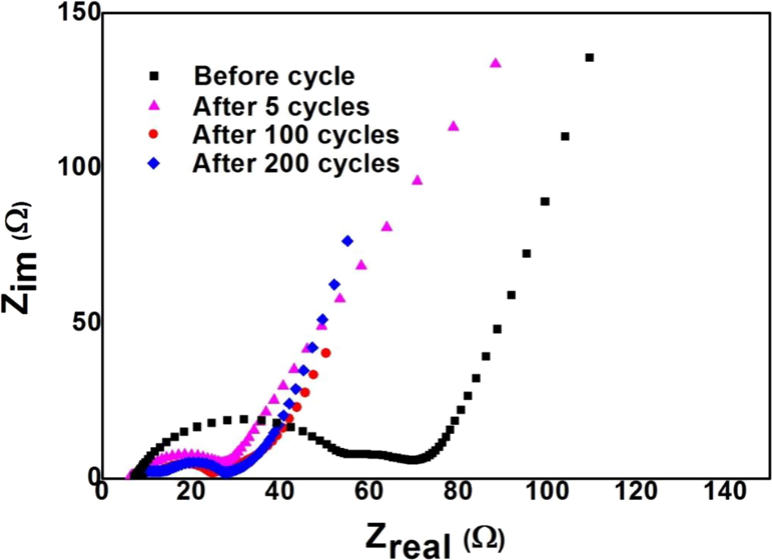
Nyquist plot of the AG electrode before the cycle and after 5, 100, and 200 cycles.

EIS measurements were performed using half-cell configuration to verify the effect of the aerogel structure on the charge transfer properties. Figure 7 shows the Nyquist plot of the AG electrode before the first cycle and after 5, 100, and 200 cycles. The Nyquist plot before cycling for the AG electrode consists of a semicircle in the high frequency region (63 Hz to 1 MHz) related to SEI resistance, a suppressed semicircle in the middle frequency range (1–63 Hz) related to charge transfer resistance, and a sloped line in the low frequency range (0.01–1 Hz) related to diffusion. After five cycles, only one semicircle is observed, probably due to the overlapping of semicircles corresponding to SEI and charge transfer. The decrease in the diameter of the semicircle after five cycles is due to the slow wetting process of the electrolyte into porous battery electrodes and the increase in the conductivity of the AG electrode after lithium-ion doping related to charging, indicating the enhanced reaction kinetics. After 100 and 200 cycles, it is observed that there is a presence of a small-suppressed semicircle of almost the same dimension in the high frequency range, corresponding to the SEI resistance and indicating a stable and thin SEI formation. However, the diameter of the semicircle in the middle frequency range is almost similar after 5, 100, and 200 cycles, indicating similar charge transfer resistance due to the thin and stable SEI layer. The charge transfer resistance does not show any significant variations after cycling over 200 times, thus exhibiting excellent cycling and rate performance.^28−30^

The actual capacity of the AG electrode is 453.8 mAh g^–1^ for the first cycle (that is slightly less than a theoretical capacity of 593 mAh g^–1^), which slightly increases to 481.6 mAh g^–1^ for the 25th cycle, obtained at a current rate of 0.1 A g^–1^. The relatively stable capacity of the AG electrode indicates the anti-pulverization structure of AG and a stable SEI layer. The formation of a stable SEI layer is also revealed from the electrochemical impedance spectroscopy results. The capacity for the electrode is highly stable as compared to the reports available in the literature.^5,31−35^ The relatively stable capacity of the AG electrode can be assigned to the anti-pulverization structure of AG and increased conductivity of the electrode. The anti-pulverization structure of the electrode is due to the nanosized particles and its high aspect ratio. It provides channels for lithium-ion intercalation and further improves electrical conductivity. This leads to effective Li-ion interactions, while the aerogel structure provides a relatively porous architecture of the electrode accommodating the volume changes of the silicon nanoparticles during lithiation–delithiation. The microstructure of aerogel wherein the silicon nanoparticles are embedded in the structure reduces the surface area of the silicon particles to be exposed to the electrolyte. Thus, minimizing the unnecessary interactions of the electrolyte with the electrode and forming a stable SEI layer further boost the performance of the electrode. These outcomes suggest the aerogel-based electrode fabrication method with changed parameters (as compared to the previous study^3^), wherein silicon nanoparticles are embedded in the aerogel structure, effectively countering the fracture of silicon particles during the lithiation process and forming a stable SEI layer and paving the pathway for key evolution in lithium-ion battery anode materials.

## 4 Conclusions

We have developed a high-performance silicon nanoparticle-based anode for LIBs using nanometer-scaled silicon particles and sodium alginate. The electrode has highly stable capacity and stable electrochemical cycling. The electrode shows 72% capacity retention up to the 500th cycle. The stable performance of the electrode is attributed to the aerogel structure, which reduces unnecessary reactions of the electrolyte with the nanoparticles and forms the stable SEI layer, as was confirmed by electrochemical impedance spectroscopy. Furthermore, the chemical synthesis and electrode manufacture process can be scalable and are compatible with large-scale slurry coating technology, having prospective for future scaling-up of high-performance anodes for lithium-ion batteries.
